# Supplementary material for: Splice-Junction-Based Mapping of Alternative Isoforms in the Human Proteome
Source: Cell Rep. Author manuscript; Available in PMC 2020 Jan 15. (PMC6961840; doi:10.1016/j.celrep.2019.11.026)
Supplement: 2 [file NIHMS1546469-supplement-2.zip › DF1/PXD010154/Prostate/Prostate_4_TUBG1_RWLGQCGNQIGFEFWK.pdf]

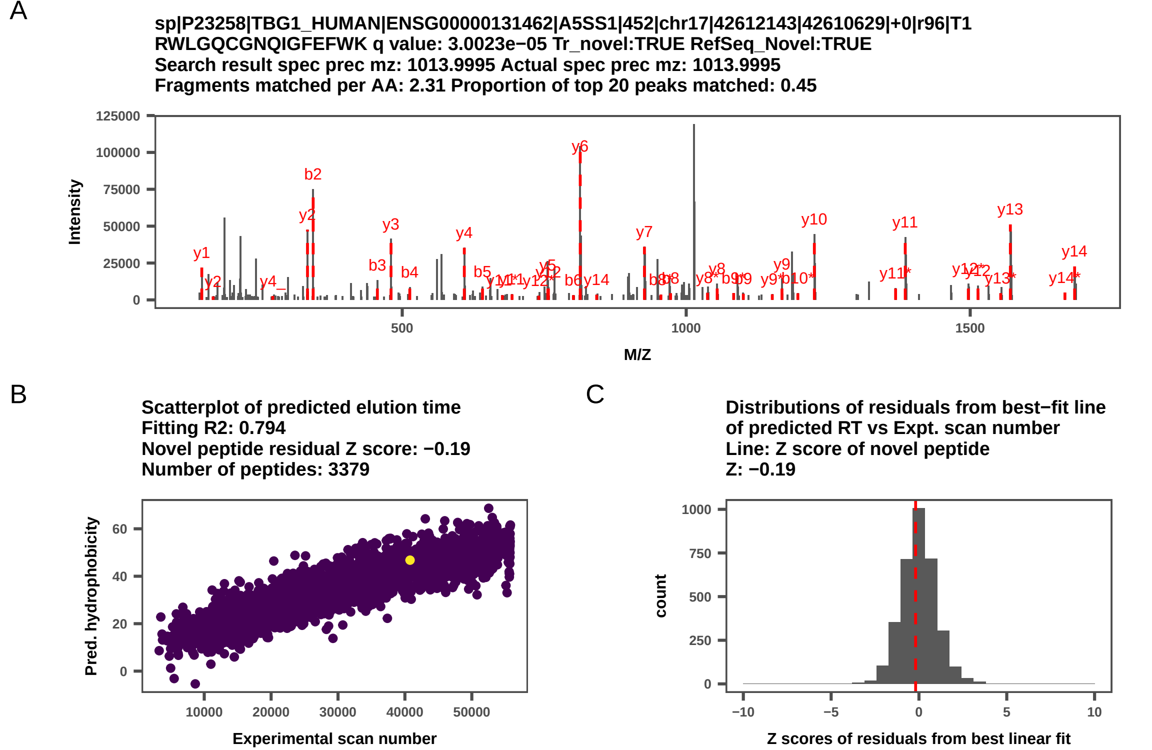
A
sp|P23258|TBG1_HUMAN|ENSG00000131462|A5SS1|452|chr17|42612143|42610629|+0|r96|T1
RWLGQCGNQIGFEFWK q value: 3.0023e−05 Tr_novel:TRUE RefSeq_Novel:TRUE
Search result spec prec mz: 1013.9995 Actual spec prec mz: 1013.9995
Fragments matched per AA: 2.31 Proportion of top 20 peaks matched: 0.45
Intensity
125000
100000
75000
50000
25000
0
y1
y2
b2
y3
b3
b4
y4
b5
y5
y6
b6
y14
y7
y8
y9
y10
y11
y11*
y13
y13*
y14
y14*
500
1000
1500
M/Z
B
Scatterplot of predicted elution time
Fitting R2: 0.794
Novel peptide residual Z score: −0.19
Number of peptides: 3379
Pred. hydrophobicity
60
40
20
0
10000
20000
30000
40000
50000
Experimental scan number
C
Distributions of residuals from best−fit line
of predicted RT vs Expt. scan number
Line: Z score of novel peptide
Z: −0.19
count
1000
750
500
250
0
−10
−5
0
5
10
Z scores of residuals from best linear fit
